# Cases of Relief from Injections into the Uterus

**Published:** 1816-10

**Authors:** 


*For the London Medical and Physical Journal.*

### *Cases of Relief from Injections into the Uterus;* by Dr. Shath.

As you expressed a wish for some cases in which the injection of the uterus, &c. had proved serviceable, the three cases following, I trust, show sufficiently the good effect which it had. Having met with a case in the obstetric line since my last, which, I think, does not frequently occur, it is at your service, if you consider it worth notice.

Case I.—March 23d, 1816.—Was called to Mrs. A—, of P—, at her full time for her first child. She was taken with great rigor; had been very sick, with frequent lax and fœtid stools; had cough and almost constant pain in the abdomen. Examined *per vaginam*, and found the os internum open about the breadth of a shilling, but rigid: gave her gt. xxv tinct. opii in a draught, after which she fell into a gentle slumber, and the diarrhœa ceased. In about six hours after, natural labour-pains came on; and she was brought to bed, in four hours after, of a stout strong child.

24th.—Every thing well, except a troublesome cough, for which she took the following mixture:

℞. Tinct. Opii gr. xij.

—— Hyocyam. ʒj.

Kali ppt. gr. xij.

Ol. Amygdal ʒiij.

Aq. ℥vij.

Syr. ʒij. M. capt. Coch. duo ter in die.

26th.—Cough better; the milk secreted in a proper quantity, but the breasts tender; the bowels confined, with some degree of thirst; lochia moderate; pulse 80 in a minute.

℞. Submur.

℞. Submur. Hyd. gr. iv.
Pulv. Jal. gr. xij.
Conf. Senæ ʒij. f. Bol. No. quatuor capt. unum 8va quaqua hora si opus fuerit.

27th.—Had proper lax stools, and every thing going on well.

30th.—Called to her late at night: found her with great soreness in the abdomen; frequent very lax offensive stools; very great thirst, with pain in the head. My house being at a distance, I could not get my apparatus for injecting the uterus that night, but ordered a clyster of milk, water, sugar, and butter, to be thrown up immediately; and to take a bolus with one grain of calomel in it, and a saline draught, with ten drops of Tr. Digitalis, to be repeated every four hours.

31st.—Early in the morning, found the abdomen considerably more swollen, and exceedingly tender, the pain in her head much increased, and frequent watery fœtid stools. Pulse 130. Had every thing ready to inject the uterus, and introduced the tube, when immediately such a gush of fœtid air was ejected as to fill the room with the scent, soon after it was injected; and she expressed great satisfaction for the ease it had given her. The milk enema was also thrown up, the abdomen fomented, and the digitalis liniment applied. In the evening, the pain, swelling, and tenderness, were very much abated; but the fever continued. Pulse 130; a dry heat; a flushed countenance; the breasts and nipples became very tender, and but little milk could be got, either by the child or nurse; great pain in the head, and not perfectly collected.

℞. Submur. Hyd. gr. iv.
Pulv. Antimon. gr. viij.
Conf. Ros. Canin. q. s. f. Bol. No. quatuor capt. unum 4ta quaq. hora cum Haust. seq.

℞. Kali ppt.

Acid. Citric āā gr. xv.

Tr. Digit. gr. x.

Syr. ʒſs.

Aq. ʒx. f. Haustus.

April 1st, 9 A. M.—Had slept a little, and the head much more composed. P. 100; had several stools, which were less offensive; the lochia moderate; the swelling of the abdomen nearly gone, but still very tender; the breasts tumid, and the nipples very sore, and very little milk; had micturated freely, and a perspiration all over the body. Injected the uterus, and ordered the clyster, fomentation, and liniment ment to be repeated once in eight hours. The bolus and draught to be taken every four hours.

2d.—Still gradually growing better, except the breasts and nipples. Bowels in a moderate state; lochia regular, and of a good colour; pulse 100; a gentle perspiration all over the body. The boluses and draughts to be taken once in six hours; the injection, &c. morning and evening.

3d.—Still gradually growing better. Pulse 90. To proceed as directed yesterday.

4th.—Still in a mending state. Pulse 90. Boluses and draughts to be taken once in eight hours; the injection, &c. once in the day.

5th.—Still mending. The bowels being confined, gave the following, and omitted the boluses and draughts:

℞. Pulv. Rhei. Magnes. āā gr. xiij.

Ol. Fœnicul. gr. ij.

Sacch. ʒſs.

Aq. ʒx. f. Laxativ.

6th.—Reported to be better.

7th.—Free from fever; the bowels confined—a laxative ut supra cum additione Pulv. Jalapii gr. vj, with which she had two motions, after which she recovered as fast as could be expected; but, the milk being in so small a quantity, and the nipples so sore, a nurse was necessary for the child.

I shall here take notice, that during the whole course of the disorder I always give my patient freely of a mucilage of the wild clary seed (i. e.) Salvia Verbeneca, which is not only grateful to the palate, but also tends to abate the inflammation, by its soft bland quality. It is made in the following manner:

℞. Sem. Salviæ Verbenecæ ʒij.

Aq. ferventis ℥iv. macera per horas duas, tunc adde Syr. Rhœados ʒij. & bene misce.

Capt. Coch. j. subinde.

Case II.—Mrs. L—, of I— House, was brought to bed of a daughter, Nov. 6, 1815, her second child,—an easy natural labour. Every thing remained well until the 10th, when she was taken with a rigor, great pain in the head, and nausea, constant retching, although she brought up but very little; bowels lax, and stools smell very offensive; lochia in a moderate quantity, but smells very fœtid; the abdomen much swollen, and very tender at the lower part. I saw her about ten A. M. about six hours after the rigor came on; her pulse then 130, a great colour in her face, a dry heat all over her body, her milk in a proper quantity, and micturated freely. Injected the uterus with milk, water, and sugar. The

The os internum was low, much dilated, but a great heat in the vagina. An enema was likewise thrown up, the abdomen fomented, and anointed with the digitalis liniment. I remained with her about an hour, when she expressed herself much relieved in the abdomen; but the pain in her head was equally as distressing as before. Ordered her to take the following:

℞. Submur. Hyd. gr. vj.
Pulv. Antimon. gr. viij.
Conf. Rosæ Gal. q. s. f. Boli No. quatuor capt. unum 6ta quaq. horâ cum quart. part. Misturarum sequentium statu effervescentiæ.

℞. Acid. Citric ʒifs.

Aq. ℥iv.

Syr. Rhæad. ʒij. sign. Mistura Febrifuga.

℞. Potas. Sub. Carbon. ʒifs.
Tr. Digital. gr. xxxx.
Aq. ℥iij. sign. Mistura Salina.

Same day, six in the evening.—Nausea lessened; pain in the head not relieved; p. 120; soreness in the abdomen still very considerable. Injected the uterus again; enema, fomentation, and liniment to be continued.

11th, 9 A. M.—Had slept three or four hours in the night; the head much relieved; a gentle perspiration all over the body; stools frequent, but not so offensive; the abdomen not so much swollen, nor so sore; p. 100. Injected the uterus again; the os internum not so hot, and, being situated so low and open, gave directions to the midwife, who was present, how to pass the injection into the uterus, and ordered it to be repeated once in eight hours, enema, &c. or oftener, in case the pain or soreness in the abdomen should increase. The boluses and mixtures to be continued as before.

12th, nine A. M.—Better in every respect; pulse 90; had slept three or four hours; the abdomen not so tender; stools frequent and fœtid. The uterus injected but once that day, but the enema, &c. repeated once in eight hours.

13th.—Reported to be better.

14th.—Found her free from all complaints, except the pain in her head, but that was not constant; bowels free; stools not offensive.

17th.—Was sent for again. The soreness and swelling in the abdomen returned; lochia stopped; bowels confined; pulse 120, and very weak; the pain in the head distressing, and talking very flighty. Immediately injected the uterus, and what was discharged was so fœtid as to annoy all in the room. The enema, fomentation, &c. also used; but, not

 being being much relieved, the uterus was injected again in three hours after, which gave great relief; ordered it to be repeated every six hours. The following was also given:

℞. Submur. Hyd. Pulv. Antim. Camphor. āā gr. iv.
Conf. Rosæ q. s. f. Boli No. quatuor sexta quaq. horâ sumend. cu Haust. seq.

℞. Potas. subcarbon. Acid. Citric āā gr. xv.

Aq. ℥x.

Æther. Nit. Spirit gr. xx.

Syr. ℥ſs. f. Haustus.

18th.—Better in every respect; pulse 100; bowels free; lochia returned.

19th.—Reported to be better. Injection, &c. to be continued.

20th.—Found her tolerable. Head free from pain; pulse 90; bowels free; lochia returned, little colour, and not offensive; bolus and draught to be taken but once in eight hours.

21st.—Reported to be comfortable; and that she began to call for food.

25th.—Found her free from fever, but very weak. Pulse 70; skin moist; bowels regular. She then began to take the Decoct. Cinchon. and soon recovered.

It is to be remarked, the milk was secreted in a proper quantity during the whole course of the fever.

Case III.—Was called to see Mrs. P—, June 18th, 1815. It was represented to me that she had an easy and natural labour on the 15th, and every thing went on well till the 17th, at night, when she began to complain of considerable pain in the abdomen, pain in her head, was very thirsty, and had passed a very troublesome night. I found the abdomen much swollen, and very tender; the body costive; lochia in small quantity; skin dry and hot; had mictu-rated freely; pulse 110. Injected the uterus immediately; soon after an enema was thrown up, the abdomen fomented and anointed, and gave her the following:

℞. Submur. Hyd. gr. iv.
Pulv. Antimon. gr. viij.
Conf. Senæ ʒij. f. Bol. No. quatuor capt. unum sexta quaq. horâ superbibend haust. infusionis Melissæ.

Saw her again in the evening: the pain and soreness in the abdomen considerably abated; had three lax stools; the head much better. Injected the uterus again, and ordered every thing to be continued as in the morning.

19th.—Better in every respect: stools frequent; lochia moderate, and not offensive; p. 100. Injected the uterus; enema, &c. to be continued.

20th.

20th.—Had a good night, and appeared to be perfectly relieved.

22d.—Every thing going on well.

It is to be observed, that I always pass the injection into the uterus the first time myself; and, if the os internum is low and open, and a midwife is present, or a nurse of sufficient adroitness, I give them instructions how to manage it, but with this particular injunction, never to press it on if it meets with any obstruction; but, when the tube enters the uterus, it generally passes on two or three inches without meeting the least resistance, and then the bladder, with the injection in it, is to be joined to the tube, and pressed up.

Case IV.—June 18th, 1816, was called to Mrs. T—, of S—, about seven months advanced in pregnancy. She was very large, had made but a very small quantity of water for the last month, but a constant desire to micturate. Introduced the catheter, and not more than a spoonful of urine was in the bladder. Threw up an enema, which, in about an hour after, was brought off, and a great quantity of fæces with it. She was exceedingly uneasy; a great tightness over the abdomen, with a very great oppression in breathing. Examined per vagina, but could not discover the os uteri, and, as she had no sleep the night before, gave her gr. xx Tr. Opii in a draught, and left her.

19th, ten A. M.—Had brought up the draught which was given last evening soon after it was taken, passed a very uneasy night, and, not having had a stool since the operation of the clyster, gave her the following:

℞. Infus. Senæ ℥x.

Mannæ Kali Tartaris. āā ʒij.

Tr. Aromat. gr. xx. f. Laxativ.

This operated in about four hours after it was taken; but it did not alleviate her distress from the load and difficulty of breathing.

20th, ten A. M.—Every thing growing worse. She could not be in any position but on her knees, with the belly resting on the bed. Examined again, and could not discover the os uteri with certainty.

21st, ten A. M.—Every complaint worse. The abdomen become of an enormous size, and the most variegated colour; very shining, and of an œdematous feel. Could not find the os uteri when she was in bed, but, the rectum being full of fæces, threw up a clyster, which soon came away, and the fæces with it. Her pulse was so quick and weak as not to be numbered. I was apprehensive that she would soon expire, or, at the least, that the uterus would soon rupture; therefore, so soon as she was moved off from the the night-stool, when in an erect position, I again endeavoured to find the os uteri, but could not, until I introduced my hand into the vagina, when I found it very high up, backward, and inclining to the left side. Having before apprised her of my intention to draw off the water, if I could with safety, I pressed a tube into the os uteri, and through the membranes, when the water began immediately to come through it, and not a drop by the side, which enabled me, in a very gradual manner, to draw off thirty beer pints of a fluid smelling more like urine than liquor amnii. She was very faint, notwithstanding that I stopped the tube very often, and had a wide bandage round the abdomen, to be drawn tight as the water was evacuated. She remained in a swooning state, with great pain in her head, for three hours, when, after taking a cup or two of coffee, she requested to be assisted to get up to make water, and made at least four ounces, saying that she was relieved beyond conception. Her pulse was then become much more firm and slow; and, in about two hours after, she was delivered of a small weak child, which lived about half an hour.

On barely reading the above case, perhaps you may be surprised at the manner of my proceeding, but I was led to suppose the cause from which all these preternatural appearances proceeded by having attended her in a very similar case before.

On the 19th of July, 1815, she being then unknown to me, I was unexpectedly sent for to her, after that she had been in labour twenty-four hours. I inquired of the midwife what she supposed the case to be, who said that she could not say, only that she had never met with the like before, as when she lay on the bed, nothing was to be felt like a child; and that she was larger than any woman she ever was with, although thin, and of a small stature. On examining, found the os internum fully dilated, the membranes loose in the vagina; and, when the pain came on, which was violent, the membranes were fully distended, strong, and reaching to the os externum. I concluded that she was with twins, and a wrong presentation; but she was strong, and in good spirits, particularly when I told her that I hoped soon to be able to relieve her; and, as soon as I conveniently could, I ruptured (or rather cut) the membranes. Such a gush of water immediately rushed out, as to cover the whole floor, and I could not feel any child: however, in about ten minutes, a pain came on, which brought down a small child (the nates presenting); and the next pain brought it into the world. It never seemed to breathe; therefore did not cut the funis till the placenta came away, which was in less than a quarter of an hour after. The placenta was larger than usual, and the membranes stronger than any I ever met with before.

*Dunstone, near Kingsbridge,*
*August* 13, 1816.

